# Event and Comment

**Published:** 1909-01-15

**Authors:** 


### DENTAL REGISTER.

**Vol. LXIII. January 15, 1909. No. 1.**

### EVENT AND COMMENT.

ALVEOLAR ABSCESS. There seems at the present time to be a disposition to treat the persistent chronic alveolar abscess, even when there is no well defined diagnosis of necrosis, by surgical procedure instead of by therapeutic remedies. This is no doubt due to the fact that surgical operations are now possible with little or no pain, and because a surgically operated wound heals much more rapidly than one disinfected by chemical cauterants or slower acting detergent remedies. There can be no question that many treatments can be greatly expedited by surgery, and also that more definite and permanent repair results from thorough surgical removal of necrotic debris. The former method of prolonged treatment of chronic alveolar abscess with phenol and iodine preparations has given way to the more agreeable antiseptics, and these seem now in a fair way to be supplanted by the surgical operation. The long and tedious treatment of pyorrhoea by medicinal reagents is uncertain in results, and as compared with the more recent surgical and prophylaxis method has scarcely anything to commend it. There is now a well grounded belief that very few cases of pyorrhoea or alveolar abscess can withstand the modern surgical treatment. The question is not now being asked as to whether pyorrhoea can be cured, but many practitioners have so strong faith in the benefits of modern methods that they are willing to drop general practice and take it up as a specialty. Surgical methods seem to have the call to-day as never before.

**DENTAL CARIES.** The question is being asked to-day as never before, is dental caries curable? It was hoped that the findings of Professor Miller as to the etiology of dental caries would very soon be followed by the discovery of the remedy. But the many years that have elapsed with no definite result would seem to indicate that either Miller's findings were incorrect or we have not yet properly interpreted them. We should however not be willing to doubt the validity of our present theories as to the cause of dental caries, any more than the medical profession should be inclined to doubt the correctness of its knowledge as to the cause of cancer or phthisis, because the remedies have nor yet been found. Our profession is making wonderful progress in technical methods and we seem likely to continue to do so, but may it not be true that we are more interested in repair methods than prevention? There seems now and then a faint glimmer of hope that some one will in the near future get a vision that will lead us to look more hopefully for some agency that will make repair work unnecessary. The oral prophylaxis propaganda, public school examinations and instruction in oral hygiene, the study of the oral secretions as possible factors in prevention, are all signs that make us hope that the coming year may throw more light on this aspect of our subject. We invite the attention of our readers to the report of the oral Hygiene committee of the National Dental Association and also the New York correspondents report on the saliva which we reprint, in part, from the *Dental Cosmos* in this issue.

**THE WEST VIRGINIA STATE DENTAL SOCIETY** held their annual meeting at Fairmont, W. Va., Oct. 14th, 15th and 16th, 1908. Dr. H. H. Harrison the President was ill at his home so the First Vice President, Dr. Chas. H. Bartlett occupied the chair. Dr. Harrison's address was read by the Secretary and Dr. Bartlett on taking the chair, delivered an able instructive paper. The first morning's session was taken up with roll call, payment of dues, discussion of needed legislation and appointment of committees. At the afternoon session Dr. Geo. H. Wilson, of Cleveland, Ohio, gave a paper (illustrated by lantern) on the Anatomical Articulation of teeth. Also a talk illustrated by charts, on Clasps, their proper and improper use and arrangement, followed by a general discussion of the subject. In the evening Dr. H. L. Ambler, of Cleveland, Ohio, gave a very interesting talk of Travels in the Orient, also illustrated by lantern slides. Thursday the 15th was entirely devoted to Clinics by the following: Dr. E. L. Kibler, Indianapolis, Ind., Porcelain Inlay, chair clinic; Dr. J. A. Libbey, Pittsburg, Pa., Immediate Root Canal Filling, chair clinic; Dr. A. C. Plant, Wheeling, W. Va., Aschers Artificial Enamel Filling, chair clinic; Dr. H. H. Myers, Pittsburg, Pa., Preparation of Cavities for Gold and Porcelain Inlays, (with models), table clinic; Dr. J. A. Libbey, , Pittsburg, Pa., Annealing Swiss Broaches, table clinic; Dr. F. L. Wright, Wheeling, W. Va., Cast Gold Inlay, chair clinic; Dr. Geo. H. Wilson, Cleveland, Ohio, Cast Aluminum Plate, table clinic; Also, models for Anatomical Articulation of Teeth, models for Clasps, table clinic; Dr. A. Earl Hennen, Wheeling, W. Va., Cast Gold Inlay, chair clinic.

At seven o'clock the Fairmont Dentists gave a banquet to the Society, which was attended by all the members, all the exhibitors, a few invited physicians and others.

Dr. John W. Storer, of Wheeling, W. Va., had been appointed toastmaster and after the feast of good things, called on the principal speaker of the evening, Mr. Lee S. Smith, of Pittsburg, Pa. Mr. Smith gave a highly entertaining talk on his travels around the world. Others were caled on by the toast-master and responded in both serious and humerous talks.

Friday morning was taken up with unfinished clinics discussions and election of officers, as follows:—President, Dr. Jas. E. Dowden, Fairmont W. Va.; Firs Vice President, Dr.

John H. McClure, Wheeling, W. Va.; Second Vice President, Dr. L. J. Walker, Grafton, W. Va.; Secretary, Dr. F. L. Wright, Wheeling, W. Va.; Treasurer, Dr. D. C. Clark, Blacksville, W. Va.

Wheeling was chosen as the next place of meeting. The time is October, 13th, 14th and 15th, 1909.

